# Conjugation-Mediated Plasmid Transfer Enables Genetic Modification of Diverse *Bacillus* Species

**DOI:** 10.1128/spectrum.03700-22

**Published:** 2023-03-28

**Authors:** Elise K. Phillips, Jordan A. Cannon, Yue Zhou, Kyle S. Bonifer, Todd B. Reynolds

**Affiliations:** a Department of Microbiology, University of Tennessee at Knoxville, Knoxville, Tennessee, USA; University of Manitoba

**Keywords:** *Bacillus*, conjugation, GFP

## Abstract

Performing genetic manipulations in *Bacillus* strains is often hindered by difficulty in identifying conditions appropriate for DNA uptake. This shortcoming limits our understanding of the functional diversity within this genus and the practical application of new strains. We have developed a simple method for increasing the genetic tractability of *Bacillus* spp. through conjugation-mediated plasmid transfer via a diaminopimelic acid (DAP) auxotrophic Escherichia coli donor strain. We observe transfer into representatives of the *Bacillus* clades *subtilis, cereus, galactosidilyticus*, and Priestia megaterium and successfully applied this protocol to 9 out of 12 strains attempted. We utilized the BioBrick 2.0 plasmids pECE743 and pECE750, as well as the CRISPR plasmid pJOE9734.1, to generate a xylose-inducible green-fluorescent protein (GFP)-expressing conjugal vector, pEP011. The use of xylose-inducible GFP ensures ease of confirming transconjugants, which enables users to quickly rule out false positives. Additionally, our plasmid backbone offers the flexibility to be used in other contexts, including transcriptional fusions and overexpression, with only a few modifications.

**IMPORTANCE** *Bacillus* species are widely used to produce proteins and to understand microbial differentiation. Unfortunately, outside a few lab strains, genetic manipulation is difficult and can prevent thorough dissection of useful phenotypes. We developed a protocol that utilizes conjugation (plasmids that initiate their own transfer) to introduce plasmids into a diverse range of *Bacillus* spp. This will facilitate a deeper study of wild isolates for both industrial and pure research uses.

## INTRODUCTION

Bacillus subtilis has become a model Gram-positive organism in part due to lab strains’ natural competence, which enables rapid genetic manipulation (1). However, as the conditions that induce competence are highly variable, identifying appropriate conditions for genetic manipulation of other isolates presents a substantial challenge. *Bacillus* spp. are widely utilized for commercial enzyme production, and novel isolates have the potential to contribute to plastic degradation (2), agricultural efficiency (3), and antibiotic production (4). Thus, expanding the repertoire of DNA introduction techniques for a broad range of bacilli will encourage further work on novel isolates.

Implementation of previously described strategies to improve transformation efficiency of *Bacillus* spp. is impeded by initially identifying reliable transformation conditions, which can be further impacted by restriction-modification systems and/or a reliance on recombination into the host genome (5). Working to improve transformation efficiency by inducing natural competence through overexpression of comK (6) and comKS (7) is effective when competence occurs, but competence conditions are highly strain specific, which limits the study of new strains.

Alternative techniques for genetic manipulation include electroporation, protoplast transformation, particle bombardment, and conjugation. Conjugation has been utilized to introduce DNA into difficult-to-transform *Bacillus* spp. and is less strain specific than some of the aforementioned methods. Triparental mating successfully generated transconjugants in two *Bacillus* clades, as well as *Paenibacillu*s, but did not produce Priestia megaterium (formerly Bacillus megaterium) transconjugants (8). An elegant conjugation-based knockout system in Priestia megaterium utilizes pasteurization as a counterselection (9), which limits the user’s study of sporulation because sporulation mutants would be unable to survive pasteurization. We sought to identify a protocol that enables conjugation into diverse bacilli and uses a simple counterselection strategy. We have developed a biparental mating system that introduces plasmid-encoded xylose-inducible GFP into *Bacillus* spp. and employs a diaminopimelic acid (DAP) auxotrophic Escherichia coli for counterselection. We observe efficient transformation in *Bacillus* clades *subtilis*, *cereus*, *galactosidilyticus*, and Priestia megaterium. This combination of E. coli donor and origin of transfer (oriT) expands Bacillus subtilis toolkits, such as the BioBrick Box plasmids (10), to additional species. The plasmid backbone, pEP011, can be easily modified to create transcriptional fusions and protein overexpression constructs for a wide range of *Bacillus* spp., further broadening its utility.

## RESULTS AND DISCUSSION

**FIG 1:**
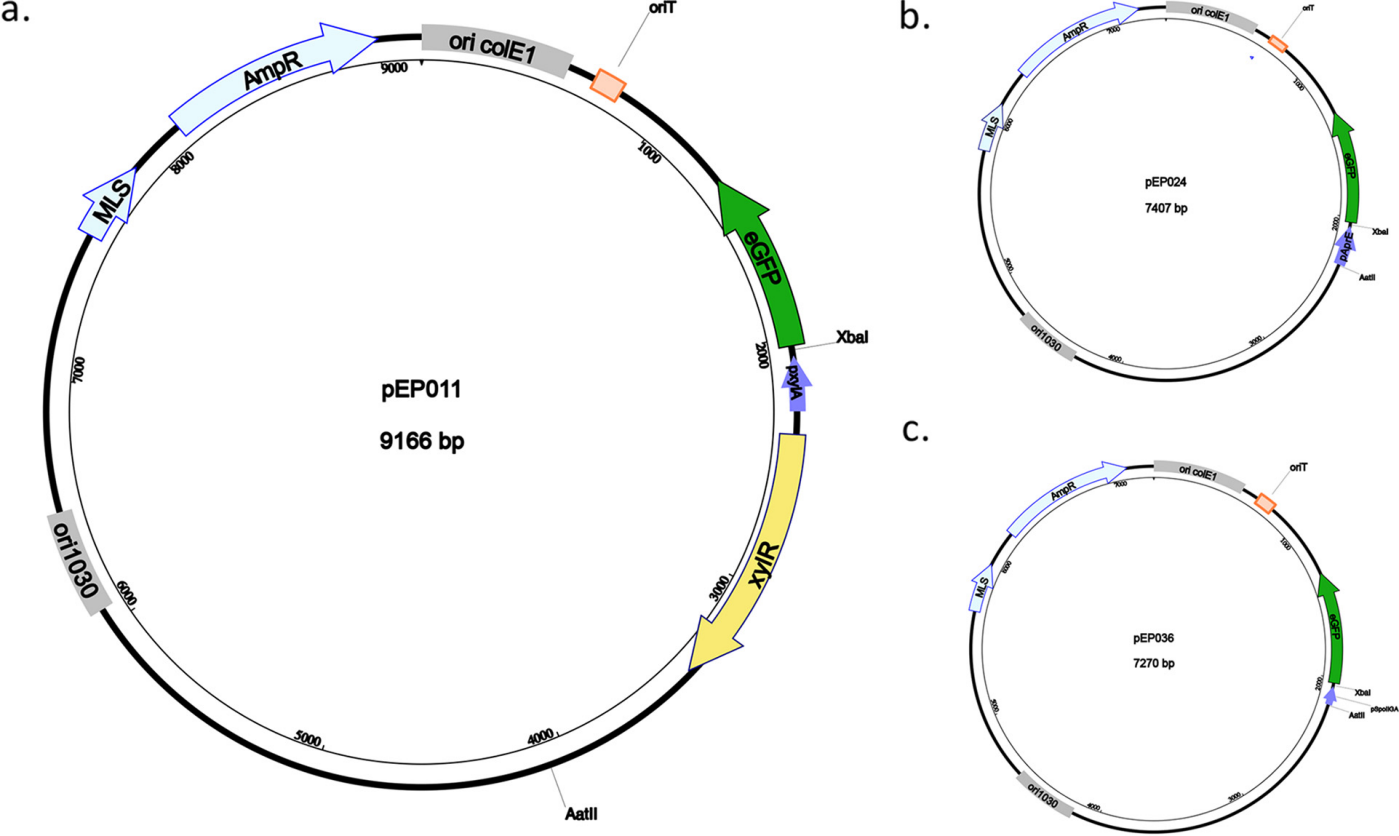
Plasmid diagrams of pEP011, pEP024, and pEP036. Light blue represents antibiotic resistance markers. MLS confers resistance to erythromycin-lincomycin for selection in *Bacillus* strains, while Amp^r^ confers resistance for selection in E. coli. Ori colE1 is the E. coli origin derived from *colE1* plasmid. Ori1030 is the replication origin for *Bacillus.* The origin of transfer (oriT) is denoted in orange. (a) pEP011. Xylose derepresses XylR (yellow), which activates *pXylA* (dark blue), thereby inducing *gfp* (green) expression. (b) pEP024. pAprE (dark blue) induces *gfp* (green) expression. (c) pEP036. pSpoIIGA (dark blue) induces *gfp* (green) expression.

We are interested in developing a strategy for genetic manipulations in the soil isolate Bacillus pumilus B12 (11) to explore the mechanism behind its ability to degrade the bioplastic poly-l-lactic acid and understand how this process is genetically regulated to improve its biotechnical applicability. Initially, many unsuccessful attempts were made to introduce DNA, including electroporation at multiple voltages (plasmids, linear single-stranded DNA, and linear double-stranded DNA), protoplasted cells, and media-induced natural competence (12–18). We then decided to switch to conjugation, a technique known for the diverse organisms that can receive DNA from a single host. We used E. coli EZ180, which has conjugation transfer genes from the plasmid RK4 integrated into its genome (19) and has been observed to act as a donor for diverse bacterial clades (20–22). The genomic integration of the RK4 transfer genes makes it possible to utilize vectors that contain a functional origin of transfer (oriT) for successful conjugation, ensuring ease of cloning and allowing the use of many potential backbones. Additionally, E. coli EZ180 has a DAP auxotrophy, which enables easy counterselection and removal of the donor bacteria from the mixed culture. To generate our conjugal vector, we cloned the *oriT* from pJoe9734.1, which another group used to conjugate CRISPR machinery into Bacillus subtilis JABs33 (23). To probe the efficiency of conjugation, a xylose-inducible GFP from pECE750 was inserted into the *oriT* containing pECE743. This plasmid, pEP011 (Fig. 1a), was transformed into the donor E. coli EZ180 strain for conjugation.

**FIG 2:**
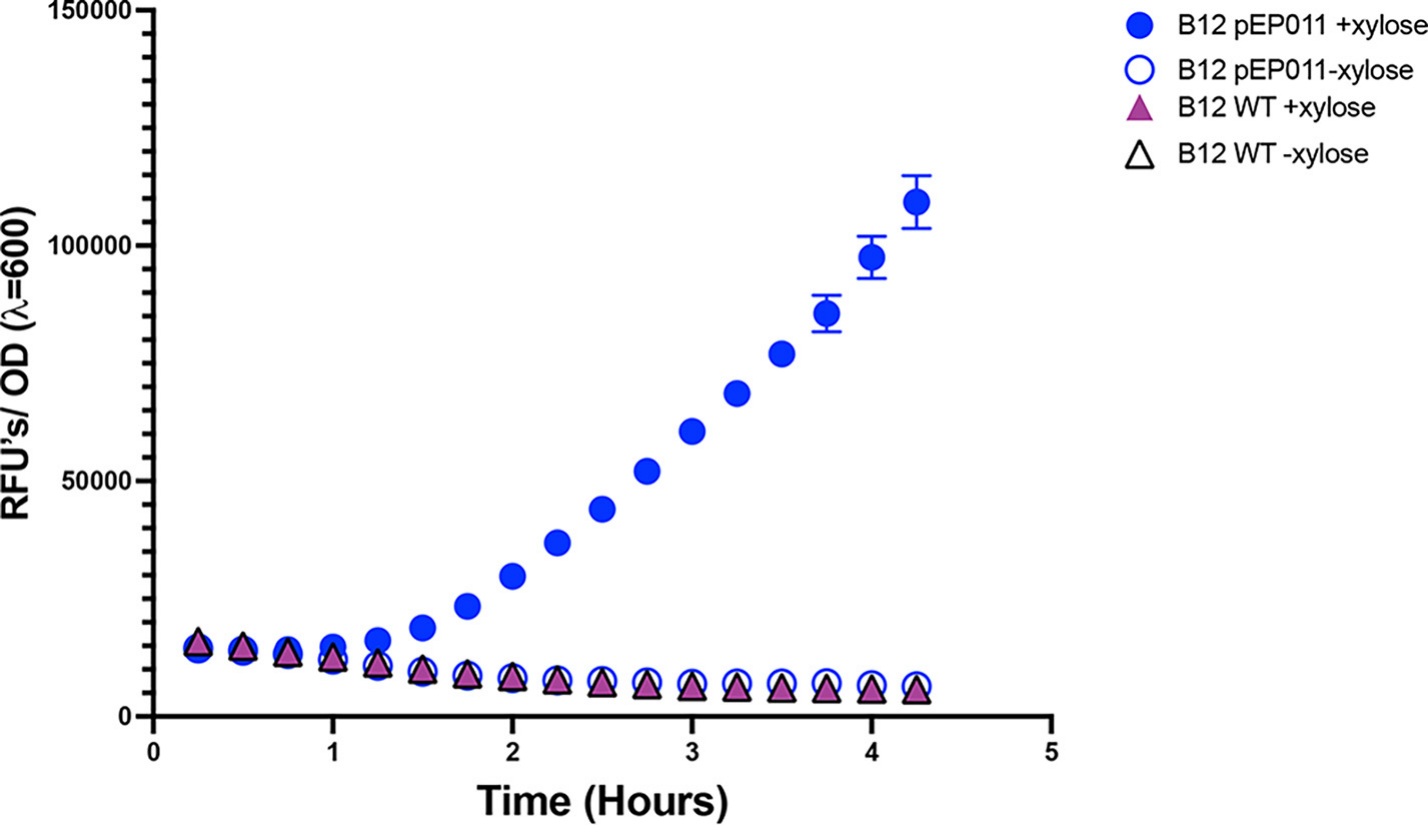
The pEP011 plasmid was successfully conjugated into Bacillus pumilus B12, and GFP was induced over time following xylose addition. Fluorescence from GFP was measured over time. The fluorescence levels were normalized to OD_600_. Error bars represent the standard error of the mean from 3 replicate wells. Two-way analysis of variance (ANOVA) with Tukey’s multiple comparisons shows that B12 with pEP011 and xylose is significantly different from the 3 control groups after 75 min.

**FIG 3:**
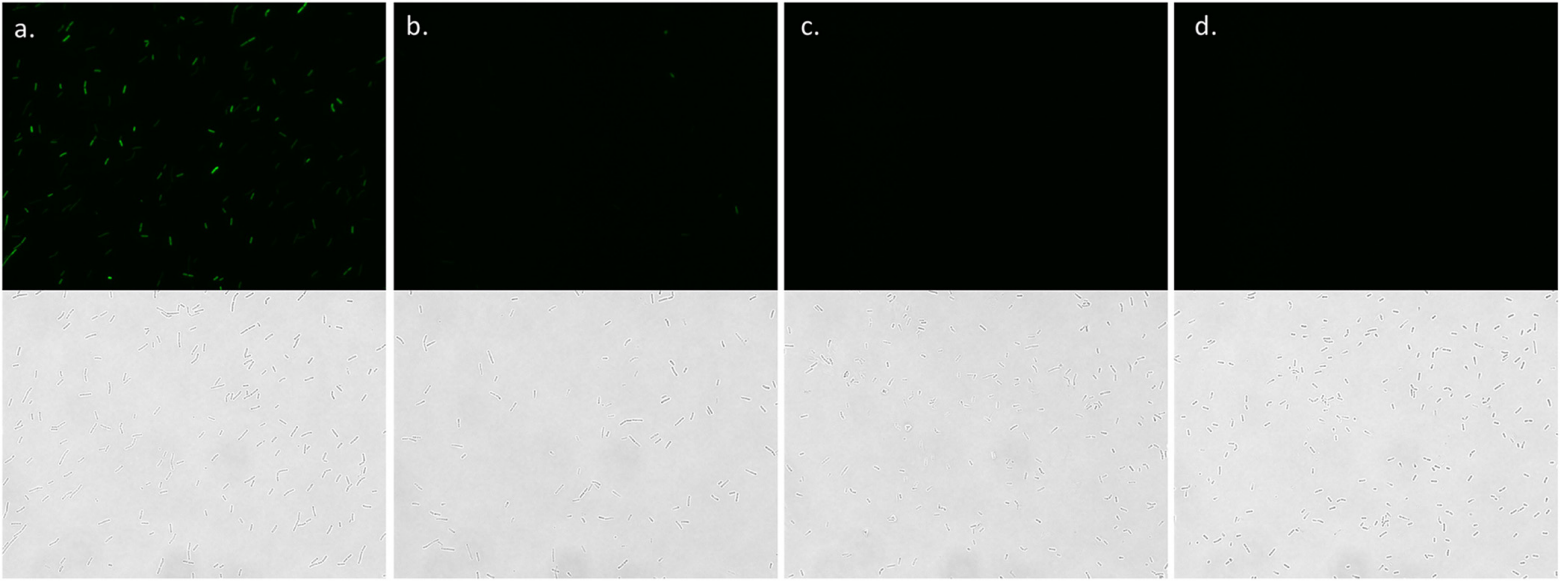
Cells with plasmid fluoresce after induction with xylose. Fluorescence (top) and bright-field (bottom) images of B12 with pEP011 (a and b) and B12 wild type (c and d) with (a and c) and without (b and d) xylose induction.

**FIG 4:**
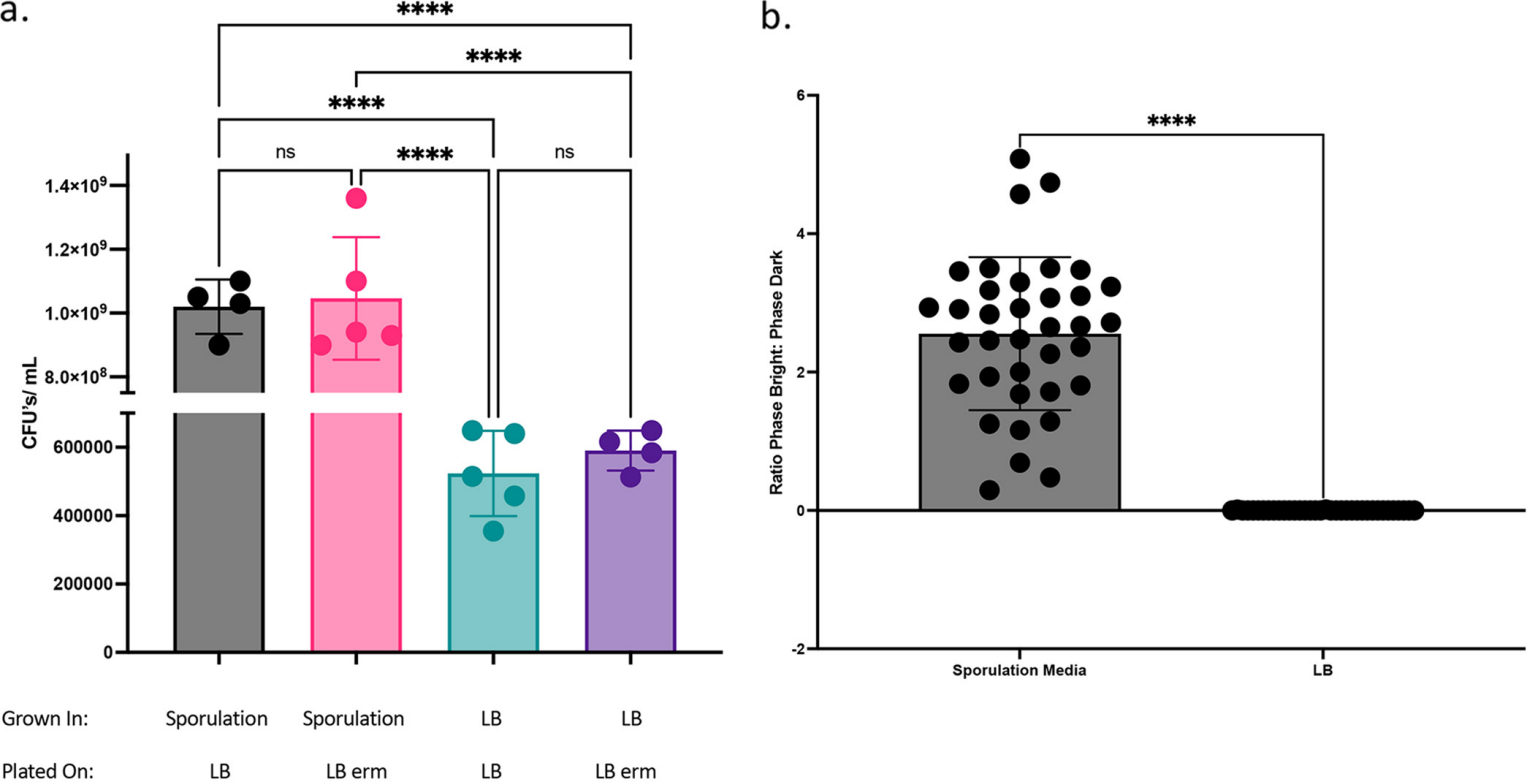
Sporulation does not impact the proportion of plasmid-containing cells. (a) CFU of heat-treated cells when grown on LB or LB erythromycin-lincomycin after 24 h of growth in either sporulation medium or LB. One-way ANOVA with Tukey’s multiple comparisons; ****, *P* < 0.0001; ns, *P* > 0.984. This is a representative figure for 1 of 3 biological replicates. (b) Proportion of phase-bright to phase-dark cells. Student’s *t* test; ****, *P* < 0.0001. This is a representative figure for 1 of 3 biological replicates.

Using this approach, we observed that conjugation between log-phase Bacillus pumilus B12 and early stationary-phase E. coli EZ180 produces numerous transconjugants. For B. pumilus B12 expressing pEP011, fluorescence above the background is statistically significantly different from control groups after 75 min of xylose induction (*P* = 0.182) and steadily increases over time when measured in a plate reader (Fig. 2). This difference is also observable via microscopy after 6 h of induction (Fig. 3). After 6 h incubation with xylose, the majority of plasmid-harboring cells are robustly fluorescent (Fig. 3a); however, without induction, only a few cells exhibit low fluorescence (Fig. 3b). This suggests that xylose induction is not very leaky. Without the plasmid, no fluorescence is observed (Fig. 3c and d), indicating that conjugation was successful with pEP011. After conjugation, we observe that the plasmid is maintained after sporulation because we obtain a similar (not statistically significantly different) number of CFUs when grown with and without erythromycin selection (Fig. 4).

**FIG 5:**
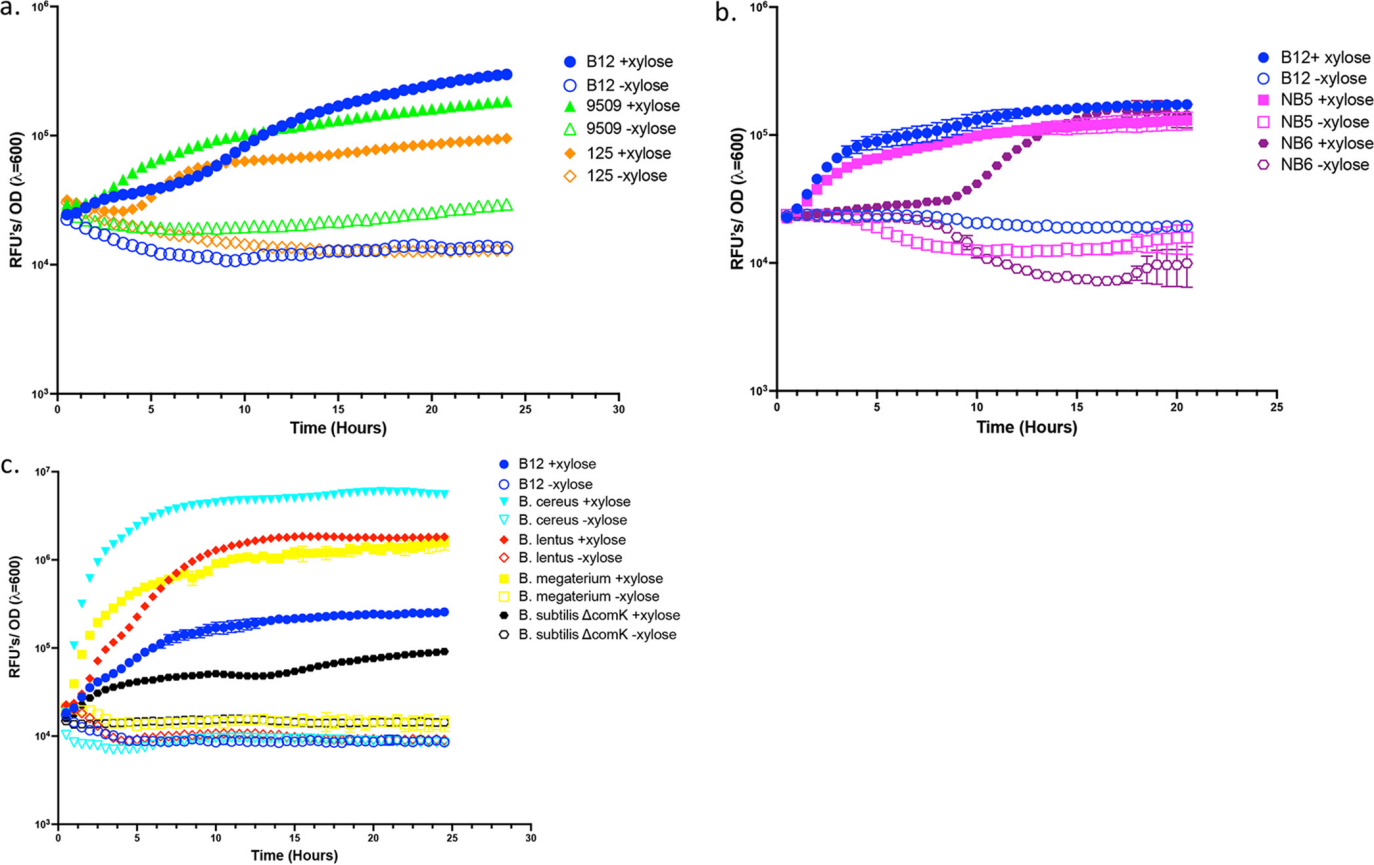
*Bacillus* spp. express GFP after xylose induction. Measurement of GFP fluorescence over 24 h normalized to optical density (λ = 600 nm). (a) Bacillus pumilus strains. (b) Environmental isolates. (c) Representatives of other *Bacillus* and closely related clades. Error bars represent standard error of the mean from 3 replicate wells.

Next, in order to measure whether this conjugation method can be used in other *Bacillus* spp., we chose representatives of 3 major *Bacillus* clades (*subtilis*, *cereus*, and *galactosidilyticus*), the closely related Prestia megaterium (24), in addition to environmental isolates in our collection that represented multiple environments (soil, aquatic, detergents). We included B. subtilis *ΔcomK* to ensure that DNA uptake occurred through conjugation rather than natural competence. We observed that multiple species within the genera *Bacillus* and *Priestia* uptake the plasmid pEP011 from E. coli EKP23 as the conjugal donor. All strains were successfully conjugated, except for Bacillus licheniformis, B. pumilus MTCC6033, and Bacillus siamensis NB9, perhaps due to differences in restriction-modification systems or antibiotic sensitivity. After xylose induction, strains expressed GFP at various levels (Fig. 5). For example, B. cereus exhibited the greatest level of GFP fluorescence, while B. subtilis *ΔcomK* exhibited the lowest expression (Fig. 5c), and environmental isolate NB6 had the longest lag before detectable GFP expression (Fig. 5b).

**FIG 6:**
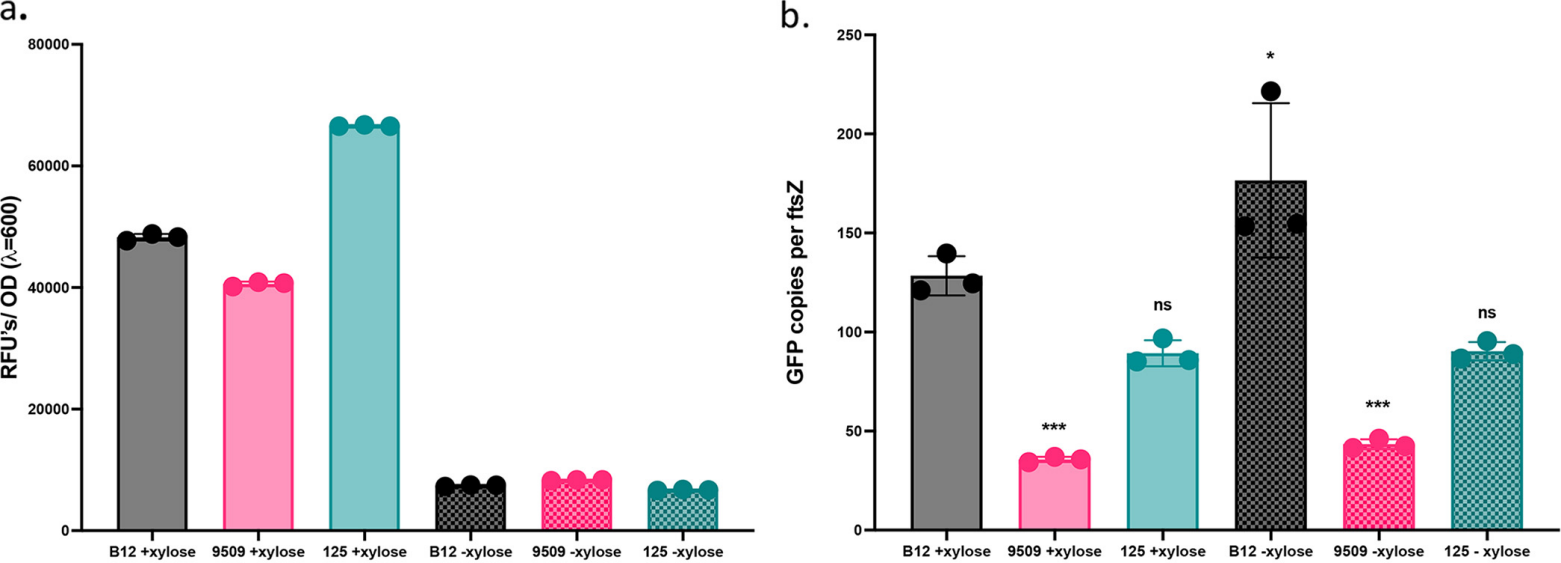
Differential expression is partially explained by plasmid copy number. (a) Normalized fluorescence after 6 h growth with or without induction. Significance determined by one-way ANOVA with Tukey’s multiple comparisons; ****, *P* < 0.0001 compared to B12 with xylose. (b) qPCR of *gfp* normalized to cellular *ftsZ* for xylose-induced and uninduced cells. Significance was determined with one-way ANOVA with Tukey’s multiple comparisons; ***, *P*, < 0.001; *, *P* < 0.05 compared to B12 with xylose. Error bars represent standard deviation. Figures are representative of two biological replicates, each with three technical replicates.

To elucidate why strains expressed GFP differentially, we performed quantitative PCR (qPCR) to quantify plasmid copy number. We normalized the amount of *gfp* to *ftsZ*, a single-copy chromosomal gene, to determine the relative copy number of the plasmid. While we observe that B. pumilus B12 fluoresces highly and robustly expresses *gfp*, this does not seem to be the case in *Bacillus* sp. strain 125, which, despite exhibiting equally high levels of fluorescence, does not have equally high relative copies of *gfp* (Fig. 6). The number of *gfp* DNA copies (plasmid number) per *ftsZ* (cell number) does not correlate with the trends in GFP fluorescence levels at 6 h. Thus, copy number of the plasmid is insufficient to explain GFP intensity alone. Strain variability in GFP fluorescence is therefore likely due to a combination of cellular attributes, including various copy numbers, strain-specific responses to the xylose-inducible promoter, and GFP expression (translation) efficiency.

**FIG 7:**
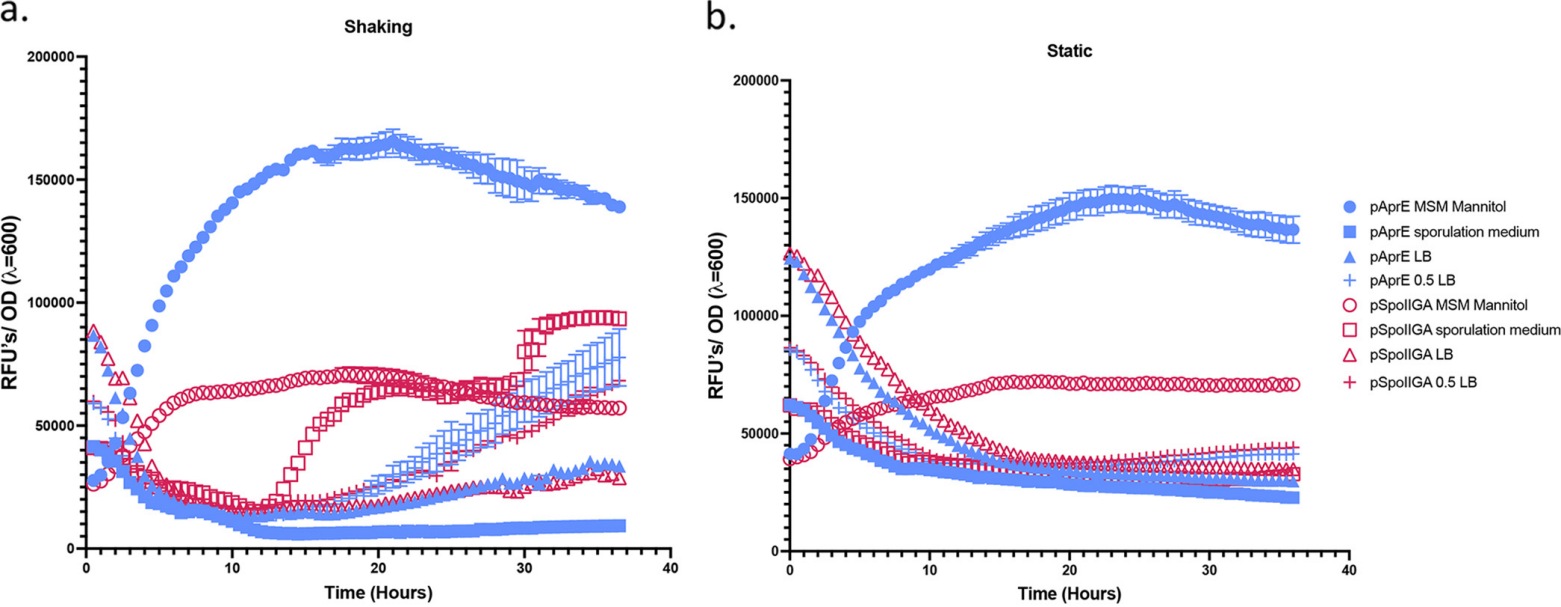
Native promoters activate GFP differentially. Measurement of GFP fluorescence over 36 h normalized to optical density (λ = 600 nm). (a) Ninety-six-well plates incubated with shaking. (b) Ninety-six-well plates incubated statically. Error bars represent standard error of the mean from 3 replicate wells.

To explore the use of this backbone as a reporter for native gene expression, we generated transcriptional fusions for two genes expressed differentially during stationary phase, *aprE* (pEP024) and *spoIIGA* (pEP036) (Fig. 1b and c). We observe that pAprE induces high GFP expression when grown in minimal media supplemented with mannitol but not in Difco sporulation media or LB. We observe moderate GFP expression induced by pSpoIIGA when grown in minimal media both statically and with shaking but only in Difco sporulation media with shaking (Fig. 7). Initial fluorescence observed in LB is due to autofluorescence of LB. The diminished relative GFP expression by pSpoIIGA is indicative of a less highly induced gene and/or a smaller subset of the population expressing the gene.

### Conclusions.

We demonstrate a single protocol that is widely applicable to *Bacillus* spp. The origin of transfer present on this backbone is promiscuous, so this protocol likely can be utilized with other plasmid-mediated gene disruption systems (25, 26). This backbone (pEP011) offers flexibility because it can be further modified to construct transcriptional fusions (pEP024 and pEP036) or overexpress genes with a strong promoter, thus making it a versatile tool. We show that biparental conjugation as a mode of plasmid introduction begins to close the gap between easy-to-genetically manipulate lab strains and difficult-to-modify environmental isolates.

## MATERIALS AND METHODS

### Bacterial growth.

Cells were grown in Luria-Bertani (LB) broth or plates with appropriate antibiotics, including ampicillin (100 μg/mL), for E. coli and erythromycin plus lincomycin (2 and 25 μg/mL, respectively) for *Bacillus* spp. E. coli EZ180 cells (used as the donor strain) were supplemented with 0.3 μM diaminopimelic acid (DAP). For fluorescence measurements with the xylose-inducible promoter, cells were grown in minimal salts medium (MSM) [per L, 1 g (NH_4_)_2_SO_4,_ 1.5 g KH_2_PO_4_, 0.5 g K_2_HPO_4_, 0.2 g MgSO_4_·7H_2_O, and 1 g NaCl] supplemented with 1% (wt/vol) fructose and 5% (vol/vol) LB. For fluorescence measurements with native promoters, MSM was supplemented with 1% (wt/vol) mannitol. All strains are listed in Table 1. Representatives of *Bacillus* clades were acquired from the *Bacillus* Genetic Stock Center (BGSC). *Bacillus* sp. strains NB5, NB6, and NB9 were isolated from a contaminated bottle of the detergent Tween 40 and identified using 16S sequencing.

**TABLE 1:** Strains used in this study

Species	Strain name	Source or reference
E. coli	EZ180	Zinser lab. Derived from WM3064 (11, 12)
E. coli	EKP23	This work
Bacillus pumilus	B12	11
*Bacillus* sp.	9509	Lebeis lab
*Bacillus* sp.	125	Lebeis lab
Bacillus pumilus	MTCC6033 (30)	Loewen lab
Bacillus subtilis	BKK10420 (ΔcomK)	BGSC
Priestia megaterium	QM B1551 (BGSC 7A16)	BGSC
Bacillus licheniformis	ATCC 14580T (BGSC 5A36T)	BGSC
Bacillus cereus	ATCC 13472 (BGSC 6A10)	BGSC
Bacillus lentus	Gibson 165 (BGSC 60A1)	BGSC
Bacillus stratosphericus ^*a*^	NB5	This work
Priestia megaterium ^*a*^	NB6	This work
Bacillus siamensis ^*a*^	NB9	This work

a Top BLAST hit with 16S sequence as of 12 August 2022.

Difco sporulation medium (27) [per L, 8 g nutrient broth no. 4 (Difco), 1 g KCl, 1 mM MgSO_4_, 1 mM Ca(NO_3_)_2_, 10 μM MnCl_2_, and 1 μM FeSO_4_] was used to induce sporulation.

### Vector construction.

Plasmid construction and maintenance were performed using NEB DH5α E. coli. pECE743 was used as the backbone plasmid because of its xylose-inducible promoter and the ability to utilize red-white screening to determine the success of GFP insertion into the plasmid (10). The origin of transfer (*oriT*) was amplified from pJOE9734.1 using primers EPO61 and EPO62 (Table 2), both of which introduced a PciI site. This fragment was cloned into the PciI site in the pECE743 plasmid (23), resulting in plasmid pEP004. The GFP open reading frame was digested from pECE750 and ligated into the XbaI and SpeI cut sites in pEP004 to create plasmid pEP011. Plasmids were transformed into calcium chloride-induced chemically competent EZ180 cells. For native promoter experiments, PCR-amplified promoter fragments were digested and inserted into pEP011 with the XbaI and AatII cut sites. Plasmids described in this study are listed in Table 3. The sequences of pEP011, pEP024, and pEP036 are found in the supplemental data.

**TABLE 2:** Primers used in this study^*a*^

Primer name	Element, cut site	Sequence
JCO70	Screening GFP insertion	ATGAGCAAAGGCGAAGAAC
JCO71	Screening GFP insertion	CAGTTCATCCATGCCATGT
EPO61	oriT, PciI	AAAA**ACATGT**GCTTGGTTTCATCAGCC
EPO62	oriT, PciI	AAAA**ACATGT**CTGCTTCGGGGTCATTATAG
EPO92	Screening transconjugants	GTCTCATGAGCGGATACATAT
EPO93	Screening transconjugants	CCATATGTTGCATCACCTT
EPO114	pAprE, AatII	AAAA**GACGTC**GCATTTCGGGTATCGAATG
EPO110	pAprE, XbaI	AAAA**TCTAGA**CTTATTTCAGAATAATCATCCG
EPO165	pSpoIIGA, AatII	AAAA**GACGTC**CGATATTCTTTCTGTTTCATACG
EPO166	pSpoIIGA, XbaI	AAAA**TCTAGA**GTTTGTACCAGTATAAAACAGTC
qEPO61	*ftsZ* (qPCR)	GACATGGTCTTTGTGACAGCC
qEPO62	*ftsZ* (qPCR)	GTGAAAGGACGAGTCACAACTC
qEPO117	*gfp* (qPCR)	CAATGCCGGAAGGCTATG
qEPO118	*gfp* (qPCR)	GCGATTGACCAGTGTATCGC
27F	16S sequencing	AGAGTTTGATCMTGGCTCAG
1522R	16S sequencing	AAGGAGGTGATCCANCCRCA

a Cut sites are shown in bold.

**TABLE 3:** Plasmids used in this study

Plasmid	Description	Source ID and reference
pECE743	XylR-P*xylA*, RFP, ori1030, Amp^r^, Mls^r^	BGSC ECE743 (10)
pECE750	GFPmut1, Cm^r^	BGSC ECE750 (10)
pJOE9734.1	oriT, Kan, pManPA-cas9	BGSC ECE722 (23)
pEP004	oriT, XylR-P*xylA,* RFP, ori1030, Amp^r^, Mls^r^	This work
pEP011	GFP, oriT, XylR-P*xylA,* ori1030, Amp^r^, Mls^r^	This work
pEP024	GFP, oriT, pAprE, ori1030, Amp^r^, Mls^r^	This work
pEP036	GFP, oriT, pSpoIIGA, ori1030, Amp^r^, Mls^r^	This work

### Conjugation.

Conjugation was performed by inoculating E. coli EKP23 (EZ180 plus pEP011) at an optical density (OD) of 0.2 in 25 mL and growing to early stationary phase (between 6 and 7 hours) in LB medium supplemented with the noncanonical amino acid DAP and ampicillin (LB-DAP-Amp) at 30°C. Simultaneously, the *Bacillus* strain to be conjugated was inoculated into 25 mL LB at an OD of 0.2 and grown to mid-logarithmic phase (OD of ~1.3) at 30°C. Each culture was spun down and resuspended in 10 mL of phosphate-buffered saline (PBS) and kept on ice until both strains reached the appropriate growth phase. An OD at 600 nm (OD_600_) of 1.0 of cells from each culture was combined in a microfuge tube, mixed by gentle pipetting, and incubated on the bench for 5 to 10 min. Three 5-μL spots of the mix were plated on LB DAP and incubated overnight (18 h) at 37°C. The three spots were scraped into 5 mL of LB erythromycin-lincomycin and recovered for 24 h at 30°C. Cells were then serially diluted and plated on LB erythromycin-lincomycin and incubated overnight at 30°C. Single colonies were streaked and screened by colony PCR with primers EPO92 and EPO93.

### Fluorescence measurements over time.

Overnight cultures of transconjugants were diluted to OD_600_ of 0.1 and then grown in minimal medium for 2.5 h at 30°C. Cultures were aliquoted into wells, and then 0.5% (wt/vol) xylose was added to appropriate wells. Fluorescence was measured using a BioTec Cytation 5 plate reader using λ of 483 nm excitation and 513 nm emission followed by an absorbance reading at λ of 600 nm while shaking at 30°C for 24 h, with reads every 15 or 30 min for 3 biological replicates.

For native promoter GFP expression, cells were inoculated into LB erythromycin-lincomycin at an OD of 0.01 and grown for 16 h overnight. Cells were washed in PBS and then inoculated into MSM mannitol at an OD of 0.2 or sporulation medium, LB, or 50% LB at an OD of 0.1. All media were supplemented with erythromycin-lincomycin as described in “Bacterial growth.” Fluorescence measurements were then taken for 36 h as described above.

### Fluorescence Microscopy.

Cells were grown as described for fluorescent measurements, but they were induced with xylose for 6 h prior to visualization via fluorescence microscopy. Cells were then imaged with a Keyence BZ-X700 fluorescence microscope using λ of 470/40 nm excitation and 525/50 emission for GFP.

### Sporulation assay.

Cells were inoculated into Difco sporulation medium with erythromycin-lincomycin or LB erythromycin-lincomycin at an OD of 0.01 and grown for 24 h at 30°C. Cells were then heat killed by incubating for 20 min at 85°C (28). Heat-killed cells were serially diluted and then plated onto LB and LB erythromycin-lincomycin. Phase-bright and phase-dark cells were quantified with phase-contrast microscopy (Olympus BX50) and Fiji image processing software.

### Plasmid copy number quantification.

To assess plasmid copy number, qPCR was used to determine the abundance of plasmid relative to a single chromosomally carried gene, *ftsZ*. Total DNA was extracted with the Winston Hoffman phenol-chloroform protocol (29) after a 6-h induction with xylose. qPCR was performed with PowerUp SYBR green master mix (Applied Biosystems, Thermo Fisher) using the manufacturer’s protocol and the primers qEPO61, 62, 117, and 118. Data were acquired and analyzed with QuantStudio3 software (Applied Biosystems). GFP expression was normalized to *ftsZ* using Livak’s method (threshold cycle [2^−ΔΔ^*^CT^*]).
