# Supplementary material for: Conjugation-Mediated Plasmid Transfer Enables Genetic Modification of Diverse Bacillus Species
Source: Microbiol Spectr. 2023 Mar 28;11(2):e03700-22. doi: 10.1128/spectrum.03700-22 (PMC10101014; doi:10.1128/spectrum.03700-22)
Supplement: Supplemental file 1 — Supplemental material. Download spectrum.03700-22-s0001.pdf, PDF file, 0.6 MB [file spectrum.03700-22-s0001.pdf]

Supplemental data-Plasmid sequences. The sequences of plasmids pEP011, pEP024, and pEP036. The pages containing the sequences of each plasmid are labeled at the top with their particular plasmid name and are in the order pEP011, pEP024, and pEP036.

```
5' ttgagatcctttttttctgcgcgtaatctgctgcttgcaaacaaaaaaaccaccgctaccagcggtggtttgtttgccgg 80
5' atcaagagctaccaactctttttccgaaggtaactggcttcagcagagcgcagataccaaatactgttcttctagtgtag 160
5' ccgtagttaggccaccacttcaagaactctgtagcaccgcctacatacctcgctctgctaatcctgttaccagtggctgc 240
5' tgccagtggcgataagtcgtgtcttaccgggttggactcaagacgatagttaccggataaggcgcagcggtcgggctgaa 320
5' cggggggttcgtgcacacagcccagcttggagcgaacgacctacaccgaactgagatacctacagcgtgagctatgagaa 400
5' agcgccacgcttcccgaagggagaaaggcggacaggtatccggtaagcggcagggtcggaacaggagagcgcacgaggga 480
5' gcttccagggggaaacgcctggtatctttatagtcctgtcgggtttcgccacctctgacttgagcgtcgatttttgtgat 560
5' gctcgtcaggggggcggagcctatggaaaaacgccagcaacgcggcctttttacggttcctggccttttgctggcctttt 640
5' gctcacatgtgcttggtttcatcagccatccgcttgccctcatctgttacgccggcggtagccggccagcctcgcagagc 720
5' aggattcccgttgagcaccgccaggtgcgaataagggacagtgaagaaggaacacccgctcgcgggtgggcctacttcac 800
5' ctatcctgcccggctgacgccgttggatacaccaaggaaagtctacacgaaccctttggcaaaatcctgtatatcgtgcg 880
5' aaaaaggatggatataccgaaaaaatcgctataatgaccccgaagcagacatgttctttcctgcgttatcccctgattct 960
5' gtggataaccgtattaccgcctttgagtgagctgataccgctcgccgcagccgaacgaccgagcgcagcgagtcagtgag 1040
5' cgaggaagcggaagagcgcccaatacgcaaaccgcctctccccgcgcgttggccgattcattaatgcagctggcacgaca 1120
5' ggtttcccgactggaaagcgggcagtgagcgcaacgcaattaatgtgagttagctcactcattaggcaccccaggcttta 1200
5' cactttatgcttccggctcgtatgttgtgtggaattgtgagcggataacaatttcacacaggaaacagctatgaccatga 1280
5' ttacgccaagcttgcatgcctgcagcggccgctactagtatttatacagttcatccatgccatgtgtaatgcctgctgct 1360
5' gtaacaaattccagcagaaccatgtgatcgcgtttttcattcgggtctttgctcagtttgctttgtgttgacagataatg 1440
5' attatccggcagcagaacagggccatctccaatcggtgtgttttgttgataatgatctgccagttgaactgagccatctt 1520
5' cgatattgtggcggattttaaagttgactttgatgccgtttttctgtttatccgccatgatatagacgttatggctatta 1600
```

```
5' taattatattccagtttgtggcccaggatatttccatcttctttaaagtcgatgcctttcagttcaatgcgattgaccag 1680
5' tgtatcgccttcaaatttaacttccgcacgcgttttgtagttgccatcatctttaaaaaagatcgttctttcttggacat 1760
5' agccttccggcattgcgcttttaaaaaaatcatgctgtttcatatggtccggatatcttgaaaagcattgaacgccatat 1840
5' gtcagtgttgtaaccagtgtcggccacggaaccggcagtttgcctgttgtgcagataaatttcagtgtcagtttgccata 1920
5' tgttgcatcaccttcgccttcgcctgaaactgaaaatttgtggccattaacgtcgccatccagttcaaccagaatcggaa 2000
5' caacgcctgtaaacagttcttcgcctttgctcattttgacctccttatctagattttttttgaattctacagatgcat 2080
5' tttatttcatatagtaagtacatcacctattagtttgttgtttaaacaaactaacttattttcatcttatataacctcgt 2160
5' cagtattttcaatatttttttagtttttatgaacacattagatataataaagggaagattcgctatgtactatgttga 2240
5' tacttaatttaaagattaaacaaatggagtggatgaagtggatatcgctgatcaaacctttgtcaaaaaagtaaatcaaa 2320
5' agttattattaaaagaaatccttaaaaattcacctatttcaagagcaaaattatctgaaatgactggattaaataaatca 2400
5' actgtctcatcacaggtaaacacgttaatgaaagaaagtatggtatttgaaataggtcaaggacaatcaagtggcggaag 2480
5' aagacctgtcatgcttgtttttaataaaaaggcaggatactccgttggaatagatgttggtgtggattatattaatggca 2560
5' ttttaacagaccttgaaggaacaatcgttcttgatcaataccgccatttggaatccaattctccagaaataacgaaagac 2640
5' attttgattgatatgattcatcactttattacgcaaatgccccaatctccgtacgggcttattggtataggtatttgcgt 2720
5' gcctggactcattgataaagatcaaaaaattgttttcactccgaactccaactggagagatattgacttaaaatcttcga 2800
5' tacaagagaagtacaatgtgcctgtttttattgaaaatgaggcaaatgctggcgcatatggagaaaaagtatttggagct 2880
5' gcaaaaaatcacgataacattatttacgtaagtatcagcacaggaatagggatcggtgttattatcaacaatcatttata 2960
5' tagaggagtaagcggcttctctggagaaatgggacatatgacaatagactttaatggtcctaaatgcagttgcggaaacc 3040
5' gaggatgctgggaattgtatgcttcagagaaggctttattaaaatctcttcagaccaaagagaaaaaactgtcctatcaa 3120
5' gatatcataaacctcgcccatctgaatgatatcggaaccttaaatgcattacaaaattttggattctatttaggaatagg 3200
```

5' ccttaccaatattctaaatactttcaacccacaagccgtaattttaagaaatagcataattgaatcgcatcctatggttt
o 3280
o
5' taaattcaatgagaagtgaagtatcatcaagggtttattcccaattaggcaatagctatgaattattgccatcttcctta
o 3360
o
5' ggacagaatgcaccggcattaggaatgtcctccattgtgattgatcattttctggacatgattacaatgtaatttttttat
o 3440
o
5' ggaatggacagctcatctttaaagatgagtttttttattctaggagtatttctgaagcaatagtgacatggcaccttctc
o 3520
o
5' atatgaaaaaggagttctaaaatagaaatctcctttttcatgtgcaaattatttttctttataacgaaaatatctaaagt
o 3600
o
5' cggccaattcactggccgtcgttttacaacgtcgtgactgggaaaaccctggcgttacccaacttaatcgccttgcagca
o 3680
o
5' catccccctttcgccagctggcgtaatagcgaagaggcccgcaccgatcgcccttcccaacagttgcgcagcctgaatgg
o 3760
o
5' cgaatggcgcctgatgcggtattttctccttacgcatctgtgcggtatttcacaccgcatatggtgcactctcagtacaa
o 3840
o
5' tctgctctgatgccgcatagttaagccagccccgacacccgccaacacccgctgacgcgccctgacgggcttgtctgctc
o 3920
o
5' ccggcatccgcttacagacaagctgtgaccgtctccgggagctgcatgtgtcagaggttttcaccgtcatcaccgaaacg
o 4000
o
5' cgcgagacgaaagggcctcgtgatacgcctatttttataggttaatgtcatgataataatggtttcttagacgtcaggtg
o 4080
o
5' gcacttttcggggaaatgtgcgcggaacccctatttgtttatttttctaaatacattcaaatatgtatccgctcatgaga
o 4160
o
5' caataaccctgataaatgcttcaataatccatcctccaaagttggagagtgagttttatgtcgcaaatattaatgtttct
o 4240
o
5' ggtgaaccttatcaaattttcgttgatttaatagaaacatagcggtaaaattagcagtaacttaatagaacggaaatgaa
o 4320
o
5' aaaagccactctcatatgctattggctaccaacctttagcgagaatgacttaatcctgtacagccatacaggacttcgac
o 4400
o
5' ttataagaggcgccaacttcaaataagttatttgccttgttttcgcgaacaaggcttattagatacacctattgtaccgt
o 4480
o
5' tactctacgaatatttcaagtagtaattactagcattgtccgttactctacgaatatttcaagtagtaattactagcatt
o 4560
o
5' gtccgttactctacgaatatttcaagtagtaattactagcattgtcatatacataataaaacggatataaaagggcgttt
o 4640
o
5' tctatacctagaagtcttgtaaatgtacagggcgtttagatatagagaacgccctttttgtgttccgttccagtggaagc
o 4720
o
5' taccactttaaaaagatggtctagtgtagccaatgcaggagagtacactcggatatcagttgtcgttgcattcaactgtc
o 4800
o

```
5'  tgacgtaagcgaggtaaaggacacaagccttgcataaaacaagcctacgggatgtaaatcctaataatgatgataaccaa
                                                                                     4880
5'  gacgttagcggcaaaaagtgttggggggttcaaaataagacatgattgtgcgactggagttaaacagttactcgtaagcgg
                                                                                     4960
5'  cgatcatgacactgattcacggctattcttgtacaagctagctttattacaaggatatgcgggttatatagcgaatcacc
                                                                                     5040
5'  cgaaagggaacggtgttgggcgtgagaaacgcaccgtacggcgcaatacaatgccaataagctatatacggacggtatag
                                                                                     5120
5'  tagttttgtaagctataaccgtttgtcgtcaatgcaaccaatctcaattcgagacctcggcatctaagccagtacgaatg
                                                                                     5200
5'  agtgggcgttttaacctcgtaaattttcaacaggggttactatgcccaaaactacattcagatttcctaacaaactcgcc
                                                                                     5280
5'  agtatgaaaaccttaagaccttaaagtcaagggatttgaaggattttaacctcgattagcaaaaaatgtagagtactgaa
                                                                                     5360
5'  gcaactaccattaactaagatagtgggggattgaggaagaatccagagctgtttaaatcaagtgaaagacaagatgaaat
                                                                                     5440
5'  taaaagaatagtgaaagataggggagtggttctctatgagaaaggaaatggctagagaacaaaggcagcggtttattgat
                                                                                     5520
5'  ctattgttagactttatggtaaagaatcctcatttatttgttaatggtacagaggatgaaagtaataatgttgttacaaa
                                                                                     5600
5'  atgtaatagtgatattaaagaggttgcggagtcatatttaactcttttatagtgagagggttaaaactaattaatatgta
                                                                                     5680
5'  ttaaggcccaatgttggaattattgtatttcactaggcaacctacttactaaaagtaagattatccattagtggatgtta
                                                                                     5760
5'  taatattgggtttttaacacaataatcatcgcctttcggtgtcgtttgatagaaaagtaaccattagcgatgaaaaagt
                                                                                     5840
5'  caatataaaaagccatccgtaaaaaacggatggcttaccgtacataggatcgttggtagggcggcgtatcctacatctct
                                                                                     5920
5'  ggtaacttacctagccaatcaaatgcttgagaacggcggttagataagcgcgtggggaacctttcccacctcaaagatcc
                                                                                     6000
5'  tatatcattattatgttactttctacaggtagtataccatgttcttatatttagtaaactccccgttagcttaacaggt
                                                                                     6080
5'  ctttgtaagcaattaaacgtccactattcaatcgtctttggattttcgcaggaccgtttttagatcgaacatagttgat
                                                                                     6160
5'  aagaacaaataaccgcttgggtccaactttatagcaattagtatatggtcatttaaaatctttaccaattcaacgctatt
                                                                                     6240
5'  aggttctttaggattttgcccgacatagtcggggtgttcaacgatatcttttatgtgcgatgaatatttttcataaatac
                                                                                     6320
5'  caggatgttgtttctttacgtgctttataaatccgggaaacatttttacatcgttagaagtgcaagtcaagttatatgta
                                                                                     6400
```

5' tctataatgatttgtggaagttttgccacaacagttggtttatttacaatctttttttattagccgtcaaatttctccc
o 6480
o
5' tcatctcgtctctttatatctttattttatcataaaggagtatttgaaccgtcgcgcgggacaggtttatgatagggata
o 6560
o
5' ttttattgaataattgatggtataagggactttcatgcttggaaagtggggattatgaattagatgcttgtccacaatat
o 6640
o
5' gttccaatgtaattaaaatttatgttcccaccttgaccaaacatcacgtccatacttaaatcgtccctcctttaataggt
o 6720
o
5' aaaatattaatttaccttaataaaaaaataatggataatagtattcgtctgaatttatataatcaggggaactattgat
o 6800
o
5' gctggggatactatttacagcggcgccatctactgatgtcgtaaaggatttgcaagataaagttatatcattgcaggatc
o 6880
o
5' atgaggtagcgtttttgaacaccacgatatctaatatgttgatccccgaagcaaacttaagagtgtgttgatagtgcagt
o 6960
o
5' atcttaaaattttgtgtataataggaattgaagttaaattagatgctaaaaatttgtaattaagaaggagggattcgtca
o 7040
o
5' tgttggtattccaaatgcgtaatgtagataaaacatctactgttttgaaacagactaaaaacagtgattacgcagataaa
o 7120
o
5' taaatacgttagattaattcctaccagtgactaatcttatgactttttaaacagataactaaaattacaaacaaatcgtt
o 7200
o
5' taacttctgtatttatttacagatgtaatcacttcaggagtaattacatgaacaaaaatataaaatattctcaaaacttt
o 7280
o
5' ttaacgagtgaaaaagtactcaaccaaataataaaacaattgaatttaaaagaaaccgataccgtttacgaaattggaac
o 7360
o
5' aggtaaagggcatttaacgacgaaactggctaaaataagtaaacaggtaacgtctattgaattagacagtcatctattca
o 7440
o
5' acttatcgtcagaaaaattaaaactgaacattcgtgtcactttaattcaccaagatattctacagtttcaattccctaac
o 7520
o
5' aaacagaggtataaaattgttgggagtattccttaccatttaagcacacaaattattaaaaaagtggtttttgaaagcca
o 7600
o
5' tgcgtctgacatctatctgattgttgaagaaggattctacaagcgtaccttggatattcaccgaacactagggttgctct
o 7680
o
5' tgcacactcaagtctcgattcagcaattgcttaagctgccagcggaatgctttcatcctaaaccaaaagtaaacagtgtc
o 7760
o
5' ttaataaaacttacccgccataccacagatgttccagataaatattggaagctatatacgtactttgtttcaaaatgggt
o 7840
o
5' caatcgagaatatcgtcaactgtttactaaaaatcagtttcatcaagcaatgaaacacgccaaagtaaacaatttaagta
o 7920
o
5' ccattacttatgagcaagtattgtctatttttaatagttatctattatttaacgggaggaaataattctatgagtcgctt
o 8000
o

5' ttttaaatttggaaagttacacgttactaaagggaatggagataaattattagatatactactgacagcttccaagaagc 8080

5' taaagaggtccctagcgcctacggggaatttggggtacattgaaaaaggaagagtatgagtattcaacatttccgtgtcg 8160

5' cccttattcccttttttgcggcattttgccttcctgtttttgctcacccagaaacgctggtgaaagtaaaagatgctgaa 8240

5' gatcagttgggtgcacgagtgggttacatcgaactggatctcaacagcggtaagatccttgagagttttcgccccgaaga 8320

5' acgttttccaatgatgagcacttttaaagttctgctatgtggcgcggtattatcccgtattgacgccgggcaagagcaac 8400

5' tcggtcgccgcatacactattctcagaatgacttggttgagtactcaccagtcacagaaaagcatcttacggatggcatg 8480

5' acagtaagagaattatgcagtgctgccataaccatgagtgataacactgcggccaacttacttctgacaacgatcggagg 8560

5' accgaaggagctaaccgcttttttgcacaacatgggggatcatgtaactcgccttgatcgttgggaaccggagctgaatg 8640

5' aagccataccaaacgacgagcgtgacaccacgatgcctgtagcaatggcaacaacgttgcgcaaactattaactggcgaa 8720

5' ctacttactctagcttcccggcaacaattaatagactggatggaggcggataaagttgcaggaccacttctgcgctcggc 8800

5' ccttccggctggctggtttattgctgataaatctggagccggtgagcgtgggtctcgcggtatcattgcagcactggggc 8880

5' cagatggtaagccctcccgtatcgtagttatctacacgacggggagtcaggcaactatggatgaacgaaatagacagatc 8960

5' gctgagataggtgcctcactgattaagcattggtaactgtcagaccaagtttactcatatatactttagattgatttaaa 9040

5' acttcatttttaatttaaaaggatctaggtgaagatcctttttgataatctcatgaccaaaatcccttaacgtgagtttt 9120

5' cgttccactgagcgtcagaccccgtagaaaagatcaaaggatcttc 9166

5' ttgagatcctttttttctgcgcgtaatctgctgcttgcaaacaaaaaaaccaccgctaccagcggtggtttgtttgccgg 80

ori colE1

5' atcaagagctaccaactctttttccgaaggtaactggcttcagcagagcgcagataccaaatactgttcttctagtgtag 160

ori colE1

AlwNI

5' ccgtagttaggccaccacttcaagaactctgtagcaccgcctacatacctcgctctgctaatcctgttaccagtggctgc 240

ori colE1

5' tgccagtggcgataagtcgtgtcttaccgggttggactcaagacgatagttaccggataaggcgcagcggtcgggctgaa 320

ori colE1

5' cggggggttcgtgcacacagcccagcttggagcgaacgacctacaccgaactgagatacctacagcgtgagctatgagaa 400

ori colE1

5' agcgccacgcttcccgaagggagaaaggcggacaggtatccggtaagcggcagggtcggaacaggagagcgcacgaggga 480

ori colE1

5' gcttccagggggaaacgcctggtatctttatagtcctgtcgggtttcgccacctctgacttgagcgtcgatttttgtgat 560

ori colE1

5' gctcgtcaggggggcggagcctatggaaaaacgccagcaacgcggcctttttacggttcctggccttttgctggcctttt 640

ori colE1

SgrAI
MreI

5' gctcacatgtgcttggtttcatcagccatccgcttgccctcatctgttacgccggcggtagccggccagcctcgcagagc 720

traJ oriT

5' aggattcccgttgagcaccgccaggtgcgaataagggacagtgaagaaggaacacccgctcgcgggtgggcctacttcac 800

oriT

5' ctatcctgcccggctgacgccgttggatacaccaaggaaagtctacacgaaccctttggcaaaatcctgtatatcgtgcg 880

oriT

5' aaaaaggatggatataccgaaaaaatcgctataatgaccccgaagcagacatgttctttcctgcgttatcccctgattct 960

RSF ori

5' gtggataaccgtattaccgcctttgagtgagctgataccgctcgccgcagccgaacgaccgagcgcagcgagtcagtgag 1040

RSF ori

SapI PvuII

5' cgaggaagcggaagagcgcccaatacgcaaaccgcctctccccgcgcgttggccgattcattaatgcagctggcacgaca 1120

RSF ori lacI

5' ggtttcccgactggaaagcgggcagtgagcgcaacgcaattaatgtgagttagctcactcattaggcaccccaggcttta 1200

lacI CAP binding site

5' cactttatgcttccggctcgtatgttgtgtggaattgtgagcggataacaatttcacacaggaaacagctatgaccatga 1280

lac promoter lac operator

PstI
NotI
HindIII SphI EagI SpeI

5' ttacgccaagcttgcatgcctgcagcggccgctactagtatttatacagttcatccatgccatgtgtaatgcctgctgct 1360

BioBrick suffix eGFP203C

5' gtaacaaattccagcagaaccatgtgatcgcgtttttcattcgggtctttgctcagtttgctttgtgttgacagataatg 1440

eGFP203C

5' attatccggcagcagaacagggccatctccaatcggtgtgttttgttgataatgatctgccagttgaactgagccatctt 1520

eGFP203C

5' cgatattgtggcggattttaaagttgactttgatgccgtttttctgtttatccgccatgatatagacgttatggctatta 1600

eGFP203C

PflMI

5' taattatattccagtttgtggcccaggatatttccatcttctttaaagtcgatgcctttcagttcaatgcgattgaccag 1680

eGFP203C

MluI

5' tgtatcgccttcaaatttaacttccgcacgcgttttgtagttgccatcatctttaaaaagatcgttctttcttggacat 1760

eGFP203C

BspEI

5' agccttccggcattgcgcttttaaaaaatcatgctgtttcatatggtccggatatcttgaaaagcattgaacgccatat 1840

eGFP203C

BtgI
5' gtcagtgttgtaaccagtgtcggccacggaaccggcagtttgcctgttgtgcagataaatttcagtgtcagtttgccata 1920
eGFP203C

MscI
5' tgttgcatcaccttcgccttcgcctgaaactgaaatttgtggccattaacgtcgccatccagttcaaccagaatcggaa 2000
eGFP203C

XbaI
5' caacgcctgtaaacagttcttcgcctttgctcattttgacctccttatctagacttatttcagaataatcatccgtagt 2080
eGFP203C pAprE

5' ctattagaatgatccgtttgattattctgtattttcagtttaatagagataggcaaataagagtagacctgaatttttgt 2160
pAprE

5' ggaagtcctagcgaaaaatagggaattaagtcgcttgggattgatggtaaaatatgtttattttatatttctaacggttt 2240
pAprE

ZraI
AatII
5' tctattaaaaaggaatcgtgctttttttctcatgatccatttgaggataaacattcgatacccgaaatgcgacgtcaggt 2320
pAprE

5' ggcacttttcggggaaatgtgcgcggaacccctatttgtttatttttctaaatacattcaaatatgtatccgctcatgag 2400
AmpR promoter

5' acaataaccctgataaatgcttcaataatccatcctccaaagttggagagtgagttttatgtcgcaaatattaatgtttc 2480
AmpR promoter

5' tggtgaaccttatcaaattttcgttgatttaatagaaacatagcggtaaaattagcagtaacttaatagaacggaaatga 2560

5' aaaaagccactctcatatgctattggctaccaacctttagcgagaatgacttaatcctgtacagccatacaggacttcga 2640

NruI
5' cttataagaggcgccaacttcaaataagttatttgccttgttttcgcgaacaaggcttattagatacacctattgtaccg 2720

5' ttactctacgaatatttcaagtagtaattactagcattgtccgttactctacgaatatttcaagtagtaattactagcat 2800

5' tgtccgttactctacgaatatttcaagtagtaattactagcattgtcatatacataataaaacggatataaaagggcgtt 2880

5' ttctatacctagaagtcttgtaaatgtacagggcgtttagatatagagaacgcccttttttgtgttccgttccagtggaag 2960

5' ctaccactttaaaaagatggtctagtgtagccaatgcaggagagtacactcggatatcagttgtcgttgcattcaactgt 3040

5' ctgacgtaagcgaggtaaaggacacaagccttgcataaaacaagcctacgggatgtaaatcctaataatgatgataacca 3120

5' agacgttagcggcaaaaagtgttgggggttcaaaataagacatgattgtgcgactggagttaaacagttactcgtaagcg 3200

NheI
BmtI

5' gcgatcatgacactgattcacggctattcttgtacaagctagctttattacaaggatatgcgggttatatagcgaatcac 3280

BsiWI

5' ccgaaagggaacggtgttgggcgtgagaaacgcaccgtacggcgcaatacaatgccaataagctatatacggacggtata 3360

5' gtagttttgtaagctataaccgtttgtcgtcaatgcaaccaatctcaattcgagacctcggcatctaagccagtacgaat 3440

AjuI' AjuI

5' gagtgggcgttttaacctcgtaaattttcaacaggggttactatgcccaaaactacattcagatttcctaacaaactcgc 3520

5' cagtatgaaaaccttaagaccttaaagtcaagggatttgaaggattttaacctcgattagcaaaaaatgtagagtactga 3600

5' agcaactaccattaactaagatagtgggggattgaggaagaatccagagctgtttaaatcaagtgaaagacaagatgaaa 3680

5' ttaaaagaatagtgaaagataggggagtggttctctatgagaaaggaaatggctagagaacaaaggcagcggtttattga 3760

5' tctattgttagactttatggtaaagaatcctcatttatttgttaatggtacagaggatgaaagtaataatgttgttacaa 3840

5' aatgtaatagtgatattaaagaggttgcggagtcatatttaactcttttatagtgagagggttaaaactaattaatatgt 3920

5' attaaggcccaatgttggaattattgtatttcactaggcaacctacttactaaaagtaagattatccattagtggatgtt 4000

5' ataatattgggtttttaacacaataatcatcgcctttcggtgtcgtttgatagaaaagtaaccattagcgatgaaaaag 4080

5' tcaatataaaaagccatccgtaaaaaacggatggcttaccgtacataggatcgttggtagggcggcgtatcctacatctc 4160

skipping-rope

5' tggtaacttacctagccaatcaaatgcttgagaacggcggttagataagcgcgtggggaacctttcccacctcaaagatc 4240

skipping-rope

PsrI' BstZ17I PsrI
5' ctatatcattattatgttactttctacaggtagtataccatgttcttatatttagtaaactccccgttagcttaacagg 4320
ori1030

5' tctttgtaagcaattaaacgtccactattcaatcgtctttggattttcgcaggaccgtttttagatcgaacatagttga 4400
ori1030

5' taagaacaaataaccgcttgggtccaactttatagcaattagtatatggtcatttaaaatctttaccaattcaacgctat 4480
ori1030

Tth111I
5' taggttctttaggattttgcccgacatagtcggggtgttcaacgatatcttttatgtgcgatgaatatttttcataaata 4560
ori1030

PfoI
5' ccaggatgttgtttctttacgtgctttataaatccgggaaacatttttacatcgttagaagtgcaagtcaagttatatgt 4640
ori1030

CspCI' CspCI
5' atctataatgatttgtggaagttttgccacaacagttggtttatttacaatctttttttattagccgtcaaatttctcc 4720
ori1030

BsmBI
5' ctcatctcgtctctttatatctttattttatcataaaggagtatttgaaccgtcgcgcggacaggtttatgatagggat 4800

5' attttattgaataattgatggtataagggactttcatgcttggaaagtggggattatgaattagatgcttgtccacaata 4880

ArsI' BmgBI ArsI EcoNI
5' tgttccaatgtaattaaaatttatgttcccaccttgaccaaacatcacgtccatacttaaatcgtccctcctttaatagg 4960

5' taaaatattaatttaccttaataaaaaaataatggataatagtattcgtctgaatttatataatcaggggaactattga 5040

5' tgctggggatactatttacagcggcgccatctactgatgtcgtaaaggatttgcaagataaagttatatcattgcaggat 5120

5' catgaggtagcgtttttgaacaccacgatatctaatatgttgatccccgaagcaaacttaagagtgtgttgatagtgcag 5200

5' tatcttaaaatttgtgtataataggaattgaagttaaattagatgctaaaaatttgtaattaagaaggagggattcgtc 5280

5' atgttggtattccaaatgcgtaatgtagataaaacatctactgttttgaaacagactaaaaacagtgattacgcagataa 5360

5' ataaatacgttagattaattcctaccagtgactaatcttatgacttttttaaacagataactaaaattacaaacaaatcgt 5440

5' ttaacttctgtatttatttacagatgtaatcacttcaggagtaattacatgaacaaaaatataaaatattctcaaaactt 5520

5' tttaacgagtgaaaaagtactcaaccaaataataaaacaattgaatttaaaagaaaccgataccgtttacgaaattggaa 5600

5' caggtaaagggcatttaacgacgaaactggctaaaataagtaaacaggtaacgtctattgaattagacagtcatctattc 5680

5' aacttatcgtcagaaaaattaaaactgaacattcgtgtcactttaattcaccaagatattctacagtttcaattccctaa 5760

5' caaacagaggtataaaattgttgggagtattccttaccatttaagcacacaaattattaaaaaagtggttttgaaagcc 5840

MLS

5' atgcgtctgacatctatctgattgttgaagaaggattctacaagcgtaccttggatattcaccgaacactagggttgctc 5920

MLS

5' ttgcacactcaagtctcgattcagcaattgcttaagctgccagcggaatgctttcatcctaaaccaaaagtaaacagtgt 6000

MLS

XcmI SnaBI

5' cttaataaaacttacccgccataccacagatgttccagataaatattggaagctatatacgtactttgtttcaaaatggg 6080

MLS

5' tcaatcgagaatatcgtcaactgtttactaaaaatcagtttcatcaagcaatgaaacacgccaaagtaaacaatttaagt 6160

MLS

5' accattacttatgagcaagtattgtctatttttaatagttatctattatttaacgggaggaaataattctatgagtcgct 6240

5' tttttaaatttggaaagttacacgttactaaagggaatggagataaattattagatatactactgacagcttccaagaag 6320

PpuMI
EcoO109I

5' ctaaagaggtccctagcgcctacggggaatttggggtacattgaaaaaggaagagtatgagtattcaacatttccgtgtc 6400

AmpR promoter AmpR

5' gcccttattcccttttttgcggcattttgccttcctgtttttgctcacccagaaacgctggtgaaagtaaaagatgctga 6480

AmpR

5' agatcagttgggtgcacgagtgggttacatcgaactggatctcaacagcggtaagatccttgagagttttcgccccgaag 6560
AmpR

5' aacgttttccaatgatgagcacttttaaagttctgctatgtggcgcggtattatcccgtattgacgccgggcaagagcaa 6640
AmpR

5' ctcggtcgccgcatacactattctcagaatgacttggttgagtactcaccagtcacagaaaagcatcttacggatggcat 6720
AmpR

PvuI
5' gacagtaagagaattatgcagtgctgccataaccatgagtgataacactgcggccaacttacttctgacaacgatcggag 6800
AmpR

5' gaccgaaggagctaaccgcttttttgcacaacatgggggatcatgtaactcgccttgatcgttgggaaccggagctgaat 6880
AmpR

FspI
5' gaagccataccaaacgacgagcgtgacaccacgatgcctgtagcaatggcaacaacgttgcgcaaactattaactggcga 6960
AmpR

5' actacttactctagcttcccggcaacaattaatagactggatggaggcggataaagttgcaggaccacttctgcgctcgg 7040
AmpR

BglI
5' cccttccggctggctggtttattgctgataaatctggagccggtgagcgtgggtctcgcggtatcattgcagcactgggg 7120
AmpR

BmrI AhdI
5' ccagatggtaagccctcccgtatcgtagttatctacacgacggggagtcaggcaactatggatgaacgaaatagacagat 7200
AmpR

5' cgctgagataggtgcctcactgattaagcattggtaactgtcagaccaagtttactcatatatactttagattgatttaa 7280
AmpR

5' aacttcatttttaatttaaaaggatctaggtgaagatcctttttgataatctcatgaccaaaatcccttaacgtgagttt 7360

5' tcgttccactgagcgtcagaccccgtagaaaagatcaaaggatcttc 7407

BmrI

5' GCTGATAAATCTGGAGCCGGTGAGCGTGGGTCTCGCGGTATCATTGCAGCACTGGGGCCAGATGGTAAGCCCTCCCGTAT 80

AhdI

5' CGTAGTTATCTACACGACGGGGAGTCAGGCAACTATGGATGAACGAAATAGACAGATCGCTGAGATAGGTGCCTCACTGA 160

5' TTAAGCATTGGTAACTGTCAGACCAAGTTTACTCATATATACTTTAGATTGATTTAAAACTTCATTTTTAATTTAAAAGG 240

5' ATCTAGGTGAAGATCCTTTTTGATAATCTCATGACCAAAATCCCTTAACGTGAGTTTTCGTTCCACTGAGCGTCAGACCC 320

5' CGTAGAAAAGATCAAAGGATCTTCTTGAGATCCTTTTTTTCTGCGCGTAATCTGCTGCTTGCAAACAAAAAAACCACCGC 400

ori colE1

5' TACCAGCGGTGGTTTGTTTGCCGGATCAAGAGCTACCAACTCTTTTTCCGAAGGTAACTGGCTTCAGCAGAGCGCAGATA 480

ori colE1

5' CCAAATACTGTTCTTCTAGTGTAGCCGTAGTTAGGCCACCACTTCAAGAACTCTGTAGCACCGCCTACATACCTCGCTCT 560

ori colE1

AlwNI

5' GCTAATCCTGTTACCAGTGGCTGCTGCCAGTGGCGATAAGTCGTGTCTTACCGGGTTGGACTCAAGACGATAGTTACCGG 640

ori colE1

5' ATAAGGCGCAGCGGTCGGGCTGAACGGGGGGTTCGTGCACACAGCCCAGCTTGGAGCGAACGACCTACACCGAACTGAGA 720

ori colE1

5' TACCTACAGCGTGAGCTATGAGAAAGCGCCACGCTTCCCGAAGGGAGAAAGGCGGACAGGTATCCGGTAAGCGGCAGGGT 800

ori colE1

BssSI

5' CGGAACAGGAGAGCGCACGAGGGAGCTTCCAGGGGGAAACGCCTGGTATCTTTATAGTCCTGTCGGGTTTCGCCACCTCT 880

ori colE1

5' GACTTGAGCGTCGATTTTTGTGATGCTCGTCAGGGGGGCGGAGCCTATGGAAAAACGCCAGCAACGCGGCCTTTTTACGG 960

ori colE1

SgrAI
MreI

5' TTCCTGGCCTTTTGCTGGCCTTTTGCTCACATGTGCTTGGTTTCATCAGCCATCCGCTTGCCCTCATCTGTTACGCCGGC 1040

5' GGTAGCCGGCCAGCCTCGCAGAGCAGGATTCCCGTTGAGCACCGCCAGGTGCGAATAAGGGACAGTGAAGAAGGAACACC 1120
oriT

5' CGCTCGCGGGTGGGCCTACTTCACCTATCCTGCCCGGCTGACGCCGTTGGATACACCAAGGAAAGTCTACACGAACCCTT 1200
oriT

5' TGGCAAAATCCTGTATATCGTGCGAAAAAGGATGGATATACCGAAAAAATCGCTATAATGACCCCGAAGCAGACATGTTC 1280

5' TTTCCTGCGTTATCCCCTGATTCTGTGGATAACCGTATTACCGCCTTTGAGTGAGCTGATACCGCTCGCCGCAGCCGAAC 1360

SapI
5' GACCGAGCGCAGCGAGTCAGTGAGCGAGGAAGCGGAAGAGCGCCCAATACGCAAACCGCCTCTCCCCGCGCGTTGGCCGA 1440

PvuII
5' TTCATTAATGCAGCTGGCACGACAGGTTTCCCGACTGGAAAGCGGGCAGTGAGCGCAACGCAATTAATGTGAGTTAGCTC 1520

5' ACTCATTAGGCACCCCAGGCTTTACACTTTATGCTTCCGGCTCGTATGTTGTGTGGAATTGTGAGCGGATAACAATTTCA 1600

PstI
NotI
HindIII SphI EagI SpeI
5' CACAGGAAACAGCTATGACCATGATTACGCCAAGCTTGCATGCCTGCAGCGGCCGCTACTAGTATTTATACAGTTCATCC 1680
eGFP

5' ATGCCATGTGTAATGCCTGCTGCTGTAACAAATTCCAGCAGAACCATGTGATCGCGTTTTTCATTCGGGTCTTTGCTCAG 1760
eGFP

5' TTTGCTTTGTGTTGACAGATAATGATTATCCGGCAGCAGAACAGGGCCATCTCCAATCGGTGTGTTTTGTTGATAATGAT 1840
eGFP

5' CTGCCAGTTGAACTGAGCCATCTTCGATATTGTGGCGGATTTTAAAGTTGACTTTGATGCCGTTTTTCTGTTTATCCGCC 1920
eGFP

PflMI
5' ATGATATAGACGTTATGGCTATTATAATTATATTCCAGTTTGTGGCCCAGGATATTTCCATCTTCTTTAAAGTCGATGCC 2000
eGFP

MluI

5' TTTCAGTTCAATGCGATTGACCAGTGTATCGCCTTCAAATTTAACTTCCGCACGCGTTTTGTAGTTGCCATCATCTTTAA 2080

eGFP

BspEI

5' AAAAGATCGTTCTTTCTTGGACATAGCCTTCCGGCATTGCGCTTTTAAAAAAATCATGCTGTTTCATATGGTCCGGATAT 2160

eGFP

BtgI

5' CTTGAAAAGCATTGAACGCCATATGTCAGTGTTGTAACCAGTGTCGGCCACGGAACCGGCAGTTTGCCTGTTGTGCAGAT 2240

eGFP

MscI

5' AAATTTCAGTGTCAGTTTGCCATATGTTGCATCACCTTCGCCTTCGCCTGAAACTGAAAATTTGTGGCCATTAACGTCGC 2320

eGFP

XbaI

5' CATCCAGTTCAACCAGAATCGGAACAACGCCTGTAAACAGTTCTTCGCCTTTGCTCATTTTTGACCTCCTTATCTAGAGT 2400

eGFP

5' TTGTACCAGTATAAAACAGTCATCGGATGGAAGTCTGTCAATTCTTGTAGGGGATGATAGAGAACTTTTGGTGGATTGTT 2480

pSpoIIGA

ZraI
SalI AatII

5' ATCGTTTTTGTCGACGTATGAAACAGAAAGAATATCGGACGTCAGGTGGCACTTTTCGGGGAAATGTGCGCGGAACCCCT 2560

pSpoIIGA

5' ATTTGTTTATTTTTCTAAATACATTCAAATATGTATCCGCTCATGAGACAATAACCCTGATAAATGCTTCAATAATCCAT 2640

5' CCTCCAAAGTTGGAGAGTGAGTTTTATGTCGCAAATATTAATGTTTCTGGTGAACCTTATCAAATTTTCGTTGATTTAAT 2720

5' AGAAACATAGCGGTAAAATTAGCAGTAACTTAATAGAACGGAAATGAAAAAAGCCACTCTCATATGCTATTGGCTACCAA 2800

5' CCTTTAGCGAGAATGACTTAATCCTGTACAGCCATACAGGACTTCGACTTATAAGAGGCGCCAACTTCAAATAAGTTATT 2880

NruI

5' TGCCTTGTTTTCGCGAACAAGGCTTATTAGATACACCTATTGTACCGTTACTCTACGAATATTTCAAGTAGTAATTACTA 2960

5' GCATTGTCCGTTACTCTACGAATATTTCAAGTAGTAATTACTAGCATTGTCCGTTACTCTACGAATATTTCAAGTAGTAA 3040

5' TTACTAGCATTGTCATATACATAATAAAACGGATATAAAAGGGCGTTTTCTATACCTAGAAGTCTTGTAAATGTACAGGG 3120

5' CGTTTAGATATAGAGAACGCCCTTTTTGTGTTCCGTTCCAGTGGAAGCTACCACTTTAAAAAGATGGTCTAGTGTAGCCA 3200

5' ATGCAGGAGAGTACACTCGGATATCAGTTGTCGTTGCATTCAACTGTCTGACGTAAGCGAGGTAAAGGACACAAGCCTTG 3280

5' CATAAAACAAGCCTACGGGATGTAAATCCTAATAATGATGATAACCAAGACGTTAGCGGCAAAAAGTGTTGGGGGTTCAA 3360

5' AATAAGACATGATTGTGCGACTGGAGTTAAACAGTTACTCGTAAGCGGCGATCATGACACTGATTCACGGCTATTCTTGT 3440

NheI BmtI

5' ACAAGCTAGCTTTATTACAAGGATATGCGGGTTATATAGCGAATCACCCGAAAGGGAACGGTGTTGGGCGTGAGAAACGC 3520

BsiWI

5' ACCGTACGGCGCAATACAATGCCAATAAGCTATATACGGACGGTATAGTAGTTTTGTAAGCTATAACCGTTTGTCGTCAA 3600

5' TGCAACCAATCTCAATTCGAGACCTCGGCATCTAAGCCAGTACGAATGAGTGGGCGTTTTAACCTCGTAAATTTTCAACA 3680

AjuI' AjuI

5' GGGGTTACTATGCCCAAAACTACATTCAGATTTCCTAACAAACTCGCCAGTATGAAAACCTTAAGACCTTAAAGTCAAGG 3760

5' GATTTGAAGGATTTTAACCTCGATTAGCAAAAAATGTAGAGTACTGAAGCAACTACCATTAACTAAGATAGTGGGGGATT 3840

5' GAGGAAGAATCCAGAGCTGTTTAAATCAAGTGAAAGACAAGATGAAATTAAAAGAATAGTGAAAGATAGGGGAGTGGTTC 3920

5' TCTATGAGAAAGGAAATGGCTAGAGAACAAAGGCAGCGGTTTATTGATCTATTGTTAGACTTTATGGTAAAGAATCCTCA 4000

5' TTTATTTGTTAATGGTACAGAGGATGAAAGTAATAATGTTGTTACAAAATGTAATAGTGATATTAAAGAGGTTGCGGAGT 4080

5' CATATTTAACTCTTTTATAGTGAGAGGGTTAAAACTAATTAATATGTATTAAGGCCCAATGTTGGAATTATTGTATTTCA 4160

5' CTAGGCAACCTACTTACTAAAAGTAAGATTATCCATTAGTGGATGTTATAATATTGGGTTTTTAACACAATAATCATCG 4240

5' CCTTTCGGTGTCGTTTGATAGAAAAGTAACCATTAGCGATGAAAAAGTCAATATAAAAAGCCATCCGTAAAAAACGGATG 4320

5' GCTTACCGTACATAGGATCGTTGGTAGGGCGGCGTATCCTACATCTCTGGTAACTTACCTAGCCAATCAAATGCTTGAGA 4400

PsrI'

5' ACGGCGGTTAGATAAGCGCGTGGGGAACCTTTCCCACCTCAAAGATCCTATATCATTATTATGTTACTTTCTACAGGTAG 4480

BstZ17I PsrI

5' TATACCATGTTCTTATATTTTAGTAAACTCCCCGTTAGCTTAACAGGTCTTTGTAAGCAATTAAACGTCCACTATTCAAT 4560

ori1030

5' CGTCTTTGGATTTTCGCAGGACCGTTTTTTAGATCGAACATAGTTGATAAGAACAAATAACCGCTTGGGTCCAACTTTAT 4640

ori1030

Tth111I

5' AGCAATTAGTATATGGTCATTTAAAATCTTTACCAATTCAACGCTATTAGGTTCTTTAGGATTTTGCCCGACATAGTCGG 4720

ori1030

5' GGTGTTCAACGATATCTTTTATGTGCGATGAATATTTTTCATAAATACCAGGATGTTGTTTCTTTACGTGCTTTATAAAT 4800

ori1030

PfoI CspCI'

5' CCGGGAAACATTTTTACATCGTTAGAAGTGCAAGTCAAGTTATATGTATCTATAATGATTTGTGGAAGTTTTGCCACAAC 4880

ori1030

CspCI BsmBI

5' AGTTGGTTTATTTACAATCTTTTTTTTATTAGCCGTCAAATTTCTCCCTCATCTCGTCTCTTTATATCTTTATTTTATCA 4960

ori1030

5' TAAAGGAGTATTTGAACCGTCGCGCGGGACAGGTTTATGATAGGGATATTTTATTGAATAATTGATGGTATAAGGGACTT 5040

ArsI'

5' TCATGCTTGGAAAGTGGGGATTATGAATTAGATGCTTGTCCACAATATGTTCCAATGTAATTAAAATTTATGTTCCCACC 5120

BmgBI ArsI EcoNI

5' TTGACCAAACATCACGTCCATACTTAAATCGTCCCTCCTTTAATAGGTAAAATATTAATTTACCTTAATAAAAAAATAAT 5200

5' GGATAATAGTATTCGTCTGAATTTATATAATCAGGGGGAACTATTGATGCTGGGGATACTATTTACAGCGGCGCCATCTA 5280

5' CTGATGTCGTAAAGGATTTGCAAGATAAAGTTATATCATTGCAGGATCATGAGGTAGCGTTTTTGAACACCACGATATCT 5360

5' AATATGTTGATCCCCGAAGCAAACTTAAGAGTGTGTTGATAGTGCAGTATCTTAAAATTTTGTGTATAATAGGAATTGAA 5440

5' GTTAAATTAGATGCTAAAAATTTGTAATTAAGAAGGAGGGATTCGTCATGTTGGTATTCCAAATGCGTAATGTAGATAAA 5520

5' ACATCTACTGTTTTGAAACAGACTAAAAACAGTGATTACGCAGATAAATAAATACGTTAGATTAATTCCTACCAGTGACT 5600

5' AATCTTATGACTTTTTAAACAGATAACTAAAATTACAAACAAATCGTTTAACTTCTGTATTTATTTACAGATGTAATCAC 5680

5' TTCAGGAGTAATTACATGAACAAAAATATAAAATATTCTCAAAACTTTTTAACGAGTGAAAAAGTACTCAACCAAATAAT 5760

5' AAAACAATTGAATTTAAAAGAAACCGATACCGTTTACGAAATTGGAACAGGTAAAGGGCATTTAACGACGAAACTGGCTA 5840

5' AAATAAGTAAACAGGTAACGTCTATTGAATTAGACAGTCATCTATTCAACTTATCGTCAGAAAAATTAAAACTGAACATT 5920

5' CGTGTCACTTTAATTCACCAAGATATTCTACAGTTTCAATTCCCTAACAAACAGAGGTATAAAATTGTTGGGAGTATTCC 6000

5' TTACCATTTAAGCACACAAATTATTAAAAAAGTGGTTTTTGAAAGCCATGCGTCTGACATCTATCTGATTGTTGAAGAAG 6080

MLS

5' GATTCTACAAGCGTACCTTGGATATTCACCGAACACTAGGGTTGCTCTTGCACACTCAAGTCTCGATTCAGCAATTGCTT 6160

MLS

5' AAGCTGCCAGCGGAATGCTTTCATCCTAAACCAAAAGTAAACAGTGTCTTAATAAAACTTACCCGCCATACCACAGATGT 6240

MLS

XcmI SnaBI

5' TCCAGATAAATATTGGAAGCTATATACGTACTTTGTTTCAAAATGGGTCAATCGAGAATATCGTCAACTGTTTACTAAAA 6320

MLS

5' ATCAGTTTCATCAAGCAATGAAACACGCCAAAGTAAACAATTTAAGTACCATTACTTATGAGCAAGTATTGTCTATTTTT 6400

MLS

5' AATAGTTATCTATTATTTAACGGGAGGAAATAATTCTATGAGTCGCTTTTTTAAATTTGGAAAGTTACACGTTACTAAAG 6480

PpuMI
EcoO109I

5' GGAATGGAGATAAATTATTAGATATACTACTGACAGCTTCCAAGAAGCTAAAGAGGTCCCTAGCGCCTACGGGGAATTTG 6560

5' GGGTACATTGAAAAAGGAAGAGTATGAGTATTCAACATTTCCGTGTCGCCCTTATTCCCTTTTTTGCGGCATTTTGCCTT 6640

DraIII

5' CCTGTTTTTGCTCACCCAGAAACGCTGGTGAAAGTAAAAGATGCTGAAGATCAGTTGGGTGCACTGAGTGGGTTACATCG 6720

5' AACTGGATCTCAACAGCGGTAAGATCCTTGAGAGTTTTCGCCCCGAAGAACGTTTTCCAATGATGAGCACTTTTAAAGTT 6800

5' CTGCTATGTGGCGCGGTATTATCCCGTATTGACGCCGGGCAAGAGCAACTCGGTCGCCGCATACACTATTCTCAGAATGA 6880

5' CTTGGTTGAGTACTCACCAGTCACAGAAAAGCATCTTACGGATGGCATGACAGTAAGAGAATTATGCAGTGCTGCCATAA 6960

PvuI

5' CCATGAGTGATAACACTGCGGCCAACTTACTTCTGACAACGATCGGAGGACCGAAGGAGCTAACCGCTTTTTTGCACAAC 7040

5' ATGGGGGATCATGTAACTCGCCTTGATCGTTGGGAACCGGAGCTGAATGAAGCCATACCAAACGACGAGCGTGACACCAC 7120

FspI

5' GATGCCTGTAGCAATGGCAACAACGTTGCGCAAACTATTAACTGGCGAACTACTTACTCTAGCTTCCCGGCAACAATTAA 7200

BglI

5' TAGACTGGATGGAGGCGGATAAAGTTGCAGGACCACTTCTGCGCTCGGCCCTTCCGGCTGGCTGGTTTATT 7271
